# Satisfaction is not all – patients' perceptions of outcome of general practice consultations, a qualitative study

**DOI:** 10.1186/1471-2296-6-43

**Published:** 2005-10-24

**Authors:** Annika Andén, Sven-Olof Andersson, Carl-Edvard Rudebeck

**Affiliations:** 1 Bergnäsets Vårdcentral, Box 80074, SE-97433 Luleå, Sweden; 2 Department of Public Health and Clinical Medicine, Family Medicine, University of Umeå, SE-90185, Umeå, Sweden; 3 Kalmar County Council, Vårdcentralen Esplanaden, SE-59330 Västervik, Sweden

## Abstract

**Background:**

Evaluation of outcome in general practice can be seen from different viewpoints. In this study we focus on the concepts patients use to describe the outcome of a consultation with a GP.

**Method:**

Patients were interviewed within a week after a consultation with a GP. The interviews were made with 20 patients in 5 focus groups and 8 individually. They were analysed with a phenomenographic research approach.

**Results:**

From the patient's perspective, the outcome of a consultation is about cure or symptom relief, understanding, confirmation, reassurance, change in self-perception and satisfaction.

**Conclusion:**

General practice consultations are often more important for patients than generally supposed. Understanding is the most basic concept.

## Background

Evaluation of outcome in general practice can be seen from different viewpoints.

Together, the patients' and the professionals' viewpoints make up the clinical perspective on outcome in medicine. Even when limited to those two, evaluation of general practice is a complex matter. Donabedian pointed out the difficulties in defining outcomes in general practice sufficiently to make them measurable [1]. To respond to the diversity and complexity of practice and its consequences, outcome research in general practice must take on many shapes.

On the whole, and according to the literature in the field, the professional perspective has dominated. Here, the measurable, bodily effects of treatment or prevention are studied and the methods include epidemiology and various forms of intervention studies, some of them fulfilling the criteria of the RCT. One example of the former is the UKDPS in which development of complications in relation to the care of the diabetic patient is studied [2], while RCTs like "Famciklovir for the treatment of acute herpes zoster" describe the effects of specific treatments [3]. If practising according to the evidence given, the GP has to rely on such research. In the individual case he does not know whether the patient is actually being treated or just taking the medicine. Outcome of treatment is here a forecast with reasonable accuracy. Intermediate outcomes, that are available in practice, such as the levels of blood pressure or HbA1c, may support the forecast, but should not be mistaken for factual outcomes.

In general practice patient-centred medicine has been increasingly important. Patient-centredness is the common denominator of a number of systematic efforts within general practice internationally, to introduce the patient as a person into the scope of the clinical method [4,5]. Patient-centredness has its roots in the Balint tradition [6] and the innovative research of Byrne and Long [7]. In the patient-centred clinical method described by Stewart et al five principal domains are included- exploring the illness experience, or expectations, the whole person, finding common ground, health promotion and enhancing the doctor-patient relationship [8].

According to the hypothesis, or hope, that it should improve health, and not just lead to good scores in psychological measures patient-centred medicine has been looked upon as an intervention. Since randomised interventions are difficult to achieve, the methods used are mainly indirect. Outcomes of patient-centred consultations have been compared to those less patient-centred. The viewpoint of the profession has decided also the evaluation of outcome of patient-centred medicine. Hard endpoints are looked for, and when psychological effects are brought within the scope, patients have to tick off predefined answering boxes to make quantification possible.

Accordingly, when studying the effects of patient-centredness, Stewart et al used the following outcome measures: patient's health measured for symptom discomfort and concern, self reported health and medical care utilization of diagnostic tests, referrals and visits to their GPs. They found that patients reported better recovery from discomfort and concern, better emotional health and fewer diagnostic tests and referrals if the patient himself perceived that the visit had been patient-centred. However this was not the case if an external judge scored audiotapes of the consultation as patient-centred [8]

Kinnersley et al inquired into whether the GPs' working style, especially patient-centredness, was related to outcome. Five generic outcomes were measured: doctor-patient agreement, patient satisfaction, resolution of symptoms, resolution of concern, and functional health. The only outcome that correlated with patient-centredness was satisfaction [9].

In the consultation, patient-centred medicine addresses the patient's view. In the extension from this should lie the interest in the patient's personal view on outcome, independent of the possible change of symptoms or disease. Studying patient satisfaction may look like doing patient-centred outcome research, but satisfaction is a multifarious concept. What are the implications of the findings of patients being satisfied? It could mean satisfaction with the doctor, the communication, the staff, the accessibility or the fulfilment of expectations. In a meta-analysis from 1988 it was noted that only 4 % of 221 studies related patient satisfaction to health outcome [10].

Jackson et al found, when evaluating satisfaction, that immediate post-visit satisfaction was heavily influenced by variables reflecting doctor-patient communication, while satisfaction within two weeks and three months was linked to symptom outcome of the consultation [11]. Clinging to patient-satisfaction, without specifying the term, or without asking patients directly, does not make the patient's experience and view become really expressed.

The limitations of patient-satisfaction as an outcome measure, has lead to the development of more nuanced protocols, such as in the PEI and PEQ instruments [12,13]. They have been developed to trace more crucial, personally-oriented outcomes than plain satisfaction, linked to patient-centredness. Enablement was greater if the doctor had been interested in the effect on the patients' life, health promotion and had had a positive approach [14].

The PEI and the PEQ spring out from the view of the profession on what should be the preferences in terms of personal outcomes. They are from the start integrated into the idea of patient-centredness. They are sensitive with regard to variations in the degree of patient-centredness in consultations. Still, there are also those outcomes that inevitably exist, beyond the doctor's aims and awareness. We find much less research in the data bases about them.

This study focuses on the patients' view of outcome as a phenomenon in its own right without looking at what actually happened in the consultation. Our aim was to draw up a systematic outline of the outcomes the patients may perceive after consultations with their GPs.

## Method

We chose to make a focus group interview study with a phenomenographic approach.

### Phenomenography

Phenomenography is a research approach originally developed when studying learning in pedagogic research [15-17]. As in other approaches of qualitative research, the aim is to describe the world as it is understood or experienced. People experience phenomena or situations in the world in qualitatively different ways, but in a limited number of ways, and the aim of phenomenography is to discern and describe such differences in a systematic way. The different ways of perceiving a phenomenon or a situation are called description categories. The description categories seen together, when they have been compared with regard to their differences and similarities, constitute what is called the outcome space.

Phenomenography has been used for health research, for instance attitudes towards physical activity among people with rheumatoid arthritis [18] or patients' long term relation to their asthma-allergy [19].

For our purpose phenomenography was a fruitful research approach. When dealing with patients, it is useful to understand how different patients can experience similar situations in different ways [20,21].

### Patients

The patients were recruited from four health centres and one after-hours general practice clinic in Luleå and Piteå in northern Sweden. They were asked by members of the staff to come for a focus group interview within a week after their latest consultation. They received written and oral information about the study.

The first step of recruitment was broad, but finally, we asked selected patients to participate, as we wanted to roughly cover the range of patients in family practice regarding age and common health problems.

Our preference for a focus group was based on the expectation that the patients in a group would inspire each other, and thus more aspects would be obtained than in a one-to-one, doctor-patient interview [22]. On the other hand we did not want a selection of patients due to the method of recruitment. As some patients hesitated to join a group interview, we added individual ones to complete the selection. We managed to recruit 5 groups with 3–6 patients in each. Twenty patients were interviewed in this way, while 8 patients were interviewed individually.

The patients were from 2–74 years, median age being 47, nineteen were women and nine were men. In the case of the two-year-old child, the mother was interviewed. The diagnostic groups were common cold (4), problems with the back and joints (9), diseases of the circulatory system (9), internal medicine diseases (4), allergies (2) dermatological problems (1), psychiatric problems (1) and health check-ups (2). Three patients had more than one problem.

Nine patients lived in the countryside and 19 in the town. Five patients had another mother tongue than Swedish. Different social classes were represented.

### Interviews

The interviews took place within a week after their latest consultation with a GP. We wanted to see them after a week, and not immediately, to give symptom change a chance to occur. We examined how they perceived the outcome of the latest consultation as this would render the freshest memories.

The group interviews were conducted by AA, assisted by a male colleague, and lasted about 1 1/2 hours. Since a pilot interview, not processed in this study, had shown that the women said very little when there were men in the group, the group interviews were carried out with men and women separately.

The individual interviews were conducted by AA and lasted 20–40 minutes.

The question introduced was: "What did you get out of your latest consultation?" An open discussion then followed which gradually was lead into a thorough discussion on the participants' experiences of their latest consultation.

In the individual interviews the interviewees became more like patients. However, in both situations the patients were outspoken about their dissatisfaction or other unpleasant facts.

### Analyses

The interviews were tape-recorded and transcribed verbatim by AA. Statements from 28 patients with 32 problems were analysed. The analysis was made in accordance with the phenomenographic analysis as described by Sjostrom with the seven steps; familiarization, compilation, condensation, grouping and classification, comparison and revision, naming of categories and description and contrastive comparison [21]. We read the transcript several times to become familiar with them. Interviews describing the outcome in similar ways were grouped together. In the accounts of the latest consultation we picked out the statements describing the outcome. These were now detached from their contexts. We categorized the statements. The description categories were compared and their content further scrutinized. The outcome space gave the overall picture of the patients' conceptions of the outcome of their latest consultation.

The Regional Ethics Committee of Umeå University approved the study.

## Results

The analysis brought forward six categories of outcome. From the patient's perspective, the outcome of a consultation is about

- cure or symptom relief

- understanding

- reassurance

- confirmation

- change in self-perception

- satisfaction

Each category contains what patients refer to as being important in their way of thinking and perception of the outcome of their latest consultation. The categories partly overlap, but are still distinct concepts.

Except for change in self-perception, the categories represent the evident needs and requests patients have when consulting. As far as the outcomes are "about" evident needs, the specific outcomes may be presented either positively or negatively; positively when the need in question is satisfied, and negatively when not.

In the following the categories will be exemplified by quotations.

### Cure or symptom relief

In this category are statements where the outcome was cure or symptom relief; either as experienced or as expected but without being obtained. This is a desired outcome but often not possible as many patients have symptoms or diseases that cannot be cured. The patients presenting this outcome often had acute or semi acute symptoms and/or disease.

The patients who had been cured did not perceive a change in self-perception, probably because they had not been confronted enough with the illness experience.

Citation: Nike (woman, age 55):"I had a pain in the elbow for several months. I work with a physiotherapist and I had been asking over and over again if she could do something. -No she said. I went to a doctor and actually he gave me a diagnosis immediately. I got treatment and I was cured. So I was very satisfied."

### Understanding

This category was relevant in all the statements about consultation outcomes. Understanding may be increased or may be a matter of frustration. All patients expressed that they wanted to "know what they had"; some wanted to know more even though they "knew what they had". The patients considered knowledge about their state to be a main outcome of the consultation. They requested knowledge of "what they had" based on their own condition, in their own circumstances and with their own understanding. An understanding might imply quite different things for different patients with a similar medical condition but may also vary over a period of time for the same patient. The name of the disease or a diagnosis was not always what they needed. Neither was the cause always a prerequisite for understanding, although the doctor may have found the explanation of the cause so obvious that there should be nothing left to wonder about.

Understanding is necessary to manage to live on with the health problems and the concern caused by them.

Citation Mari (woman age 46):"A diagnosis for me is completely unessential. What I want is that they realize why I have pain. So I can get rid of it."

Patients, who felt that they had not acquired an understanding of their condition, were dissatisfied with the outcome even if they had been cured.

Lejla (woman, age 39):"But I mean, just relieving the pain does not help, you also have to know why you have it. It doesn't help just to be relieved you must know in some way how to handle it to be able to prevent more pain."

Understanding must not be mixed up with explanation because an explanation in the abstract that does not respond to one's experienced needs is of no benefit.

Citation: Siri (woman 18):"They never explained why I got this skin infection, they just said it is some staphylo- or strepto-something."

In other situations, though, information about the condition and nothing more, may lead to a new understanding, which was then the outcome of the consultation.

Citation Erik (man age 63):"I have some stuff that goes from my kidneys into the blood, I don't know the name of it, I don't have to because I am not the doctor, but it could get worse if I stopped taking my antihypertensive, it could be dangerous for me and it would really be rotten to get kidney problems."

### Confirmation

The patients had often had thoughts about their symptoms /disease before the consultation, and maybe also fantasies of how it could develop. The GP had observed and listened, added some tests in a few cases, but had then not taken any action beyond confirmation. An outcome for some patients was that their fears or fantasies were confirmed or unconfirmed.

Citation Anette (woman 33):"-So I asked that doctor -Do I have fibromyalgia? Because I had been thinking and wondering. – Yes she said that has been established. – Yes thank you, then I know, I said. I got to know this half a year ago when my ordinary doctor was not here, and I had had this pain for four and a half years but I had never got round to asking before." (This citation referred to a consultation half a year earlier)

A confirmation of a disease, even when serious, is at the same time a confirmation of an experience, and therefore not only negative. The prevailing uncertainty when the doctor does not know, or does not respond to the worrying experience, may however in itself be a torment. Outcomes of this nature, presented by patients who were thrown into uncertainty, were also placed in this category. They had been referred, and were waiting for further treatment or assessments. They did not know what they could expect and were left in a state of confusion. They did not express satisfaction, dissatisfaction or any other feelings with regard to the consultation. The uncertainty of the situation dominated.

Nils (man, age 73):"You will see then, you know, if it is the kidneys that are.... The function of the kidneys has been a little poor .... I get so tired all of a sudden and that's not good...I am not exactly ready to die yet...But you won't know from one day to another. I have to find out the reason for my being so tired. I mostly want to lie down, but you can't lie down all the time. You have to keep moving.

For others the confirmation dealt with the fact that they were doing the right thing. Their judgement was recognized by the doctor.

Citation Gudrun (woman 49):"She (the GP) examined me very well. I said I had been seeing a physiotherapist, and she told me to go on with the exercise that I had been taught. I told her I had been taking painkillers, aspirin and paracetamol, and she told me to go on with that, and to take it easy."

For some patients an assessment or information about their condition was the only outcome of the consultation. They had diseases that did not make them disabled but rather were to be considered as risk factors. Without having been worried they had got it confirmed that everything was well.

Citation Johnny (man age 62):"Now I have good tests on everything, everything was perfect. That was the good experience of my visit."

Some patients perceived lack of confirmation although they had expected it.

Citation Cecilia (woman, age 36):"I came to this doctor and told him about all the strange allergies I had had this last week, and showed him my wrist, that all of a sudden had become so swollen. So, he said you must have a sprain, and he gave me naproxene. I felt so misunderstood and I was so angry, that I went home and took the cortisone I had got the other day."

### Reassurance

Some patients, who had been worried before the consultation, perceived reassurance as an outcome. Their fears were not confirmed. Once the cause of worry had been refuted, the worry itself was much diminished and almost forgotten.

A reassurance could be both explicit and implicit. An assessment saying that there is nothing dangerous going on is an explicit outcome.

Citation Nils (man 73):"I had felt extra beats from my heart and that made my pulse jump. I thought I would maybe need a pacemaker but my Doctor said I did not. It will probably disappear by itself. It was a good thing that I don't need a pacemaker."

A reassurance can also be implicit. Getting a diagnosis or an explanation of symptoms implies that it is not another, dangerous disease. The fear did not have to be mentioned. The fear of cancer was seldom openly expressed but often between the lines.

Citation Johnny (man 63):"Now I have good tests on everything... That was the good experience of my visit. I was not worried. But people around, they die. You are at the age for prostatic cancer.

Often reassurance was seen together with confirmation, especially when a worry had been confuted. But they could also be separate. Patients who had changed their image of themselves perceived confirmation but did not mention reassurance.

### Change in self-perception – accepting the reality of the body

In this category we find statements from patients who had had the symptoms or the disease for a long time and now had reached the understanding that it would persist. The consultation had been the last in a row where earlier consultations had gradually prepared for a more definite change in self-perception. In this very consultation knowledge had turned into acceptance of the reality of the body and now they were ready to face their future searching for strategies to handle their situation and their lives. The illness/disease did not change but they were satisfied with the outcome.

This outcome was seen in some form in a fourth of the patients' descriptions.

Citation Hedvig (woman age 59):"It's something neurological...it's something in the brain you know...you can't know for sure what is actually the cause of it...How do you get on with your life after this? The consultation before last was about the whole of me and everything that concerns me... The last time I came here there were three ready suggestions for me; thus the sadness, but also relief. I'm not at all disappointed with that doctor or so, the sadness is about other things – about life."

### Satisfaction

A manifestation of satisfaction or dissatisfaction was to be found in most of the patients' statements. It was rather expressed as positive or negative assessments of consultation outcome than as an explicit degree of satisfaction. Statements of satisfaction or dissatisfaction often functioned as a summing up or conclusion of the patient's evaluation of the outcome. Satisfaction was never the main outcome.

The patients were satisfied when they had acquired an understanding of the condition. Patients who "knew what they had" were satisfied even though they were not relieved or cured.

Citation Curt (man age 74):"Now the last time my blood pressure had gone down so it was just 170 over 70 and that was good in my case, it had gone down.

Patients who did not know what they had were dissatisfied even if they had been cured.

Citation Mia (woman age 36):"I didn't even get to know what I had. I was so angry with myself- why had I not asked? I had to call back to the nurse and ask and she said you have tonsillitis."

Maja (woman age 20):"I went to the doctor and he bent my knees back and forth and pressed them a little, and then he gave me a prescription for pain killers. But I wanted to know what it might be. He could not answer, because he did not know. I was very dissatisfied with going there. I had realized that myself, that I had pain and needed pain killers but I wanted to know what it was. If he couldn't help me, I think it was his duty to send me to a specialist."

All the patients who had acquired an understanding were satisfied. Some were satisfied if they had received confirmation but not an understanding.

All the patients who had not acquired an understanding or received confirmation were dissatisfied. They were dissatisfied even if they had been cured. They did not feel reassured.

Patients who were dissatisfied felt that they had not been seen or heard during the consultation.

They were mostly women whose mother tongue was different from the doctor's.

## Discussion

The patients' perceptions of the outcome of their latest consultation with a GP can be described with the concepts; cure or symptom relief, understanding, reassurance, confirmation, change in self-perception and satisfaction.

The analysis illuminates a spectrum of categories- meanings contained within what is perceived as outcome by patients in general practice. Some are of course self evident and also well researched, while others probably are less well recognized, or less recognized as to their possible importance.

Satisfaction has been measured in many ways. Two satisfaction instruments are CSQ [23] and MISS [24]. They have the main focus on the consultation situations but few items on outcome.

Satisfaction is necessary as a part of an evaluation but not enough on its own.

Howie et al developed the Patient Enablement Instrument, PEI, with questions immediately after the consultation whether the patient could understand his illness, and cope with illness and life in a better way [12]. This covers the concept of understanding and is close to the concept "a change in the perception of oneself".

The PEQ questionnaire is broader and has focused on the patients' experiences of the consultation in terms of communication, emotions, outcome similar to the PEI questions, barriers and auxiliary staff [13].

There are several validated instruments to measure change of health or symptoms. The SF-36 [25], EuroQol [26]and MYMOP [27] are such instruments.

What emerges as an important finding, and which is not so much an item of other outcome studies, is that patients do not assess outcome predominately as a change of symptoms. Outcome is a change within a context, that embraces the person, the body as an aspect of the person, and the person's understanding of what is going on in his/her own, physical body.

They seldom mentioned prescriptions or sick-listing, and they did not regard such measures as outcomes.

The main position of understanding is in accordance with the goals for patient-centred care, but while understanding is there, above all an aspect of "finding common ground", our findings suggest that it should be regarded as an end in itself.

Our results indicate that outcomes of consultations, from the patients' point of view, to a great extent concern how to deal with life changes caused by ill health. In the first place this may be accomplished through increased knowledge and understanding, in the second through cure or relief and in the third through the acceptance of change and finding coping strategies. This is in accordance with Helen Ekströms recent thesis, "Keeping my ways of being", where she found that the patients who go through a bodily change finally reach a reappraisal of themselves [28].

One definition of health, which is relevant in this context, is a "home-likeness" in the world [29]. The patients who had experienced a change in their self-perception caused by disease had lost that feeling, but after this latest consultation they were on their way home again.

We believe that the overall, systematic picture of perceived outcome has relevance in itself. Being aware of the possible range of outcomes, in the consultation and in the longer term, the GP may trace the effects of his/her own actions in a more sensitive way. The signs should be there in the way patients talk, act and react. It is not necessary that all become matters of open discussion. Some of the outcomes, like confirmation or change in self-perception, may not even be a possible request for the patient. Still they should be parts of a valid doctor-patient relation. In recognizing the implicit, that which can not be verbalized, our findings may be a valuable addition where the spirit of explicitness, so eloquently argued for by the protagonists of patient-centredness, falls short.

## Limitations of the study

The selection was purposeful in relation to the aim of the research [30]. The 28 patients represented both sexes, all ages, symptoms and diseases common in general practice and different social circumstances. Still we cannot maintain that the outcome space is complete. Other outcomes might have been described if more patients had been interviewed.

Adding individual interviews for those who preferred meant that it was not only patients who were positive to group participation that were included.

The focus group interviews were richer in content, but the statements were interwoven and had to be unravelled. In the individual interviews the patients took a more passive role. There was one essential difference between the content of the two types of interviews, since a change in the perception of oneself was only described in the focus groups. The other categories were represented in both sorts of interviews. This strengthens the presupposition that the patients were more free in group interviews.

The impact of the consultation works over a period of time. The interviews were made within a week, which might be too short a period to see some outcome for some patients, but with a longer interval other patients could have forgotten.

## Conclusions of the study

- The categories of the perceptions of the patients' outcome that we have described have been investigated and measured to some extent in earlier studies, but here the picture is more complete.

- The results imply that general practice consultations often are more important for patients than generally supposed. The most radical outcome is a change in self- perception, which is a big thing in the individual's world.

- Understanding is the most basic outcome, being an aspect of all the others.

- Cure, or remedy, that many doctors regard as the most self-evident outcome, is quite often of limited importance.

- Satisfaction relates to all the other categories, but is first and foremost a function of the understanding.

- The predicament of the patient has a major impact on what turns out to be the outcome. Seemingly small contributions from the doctor, like the pure confirmation of the state of matters, may become great as to their effects.

## Competing interests

The author(s) declare that they have no competing interests.

## Authors' contributions

AA made the interviews and transcribed them.

AA and SOA made the design of the study.

AA and SOA and CER made the analyses and wrote the article.

## Pre-publication history

The pre-publication history for this paper can be accessed here:


